# Targeted Mutational Profiling and a Powerful Risk Score as Additional Tools for the Diagnosis of Papillary Thyroid Cancer

**DOI:** 10.1007/s12253-019-00772-4

**Published:** 2019-11-22

**Authors:** Barbara Kocsis-Deák, Kristóf Árvai, Bernadett Balla, Bálint Tóbiás, Andrea Kohánka, Balázs Járay, János Horányi, János Podani, István Takács, Zsuzsanna Putz, János Kósa, Péter Lakatos

**Affiliations:** 1 1st Department of Internal Medicine, Semmelweis University, Korányi Sándor Street 2/a, Budapest, 1083 Hungary; 2 PentaCore Laboratory, Budapest, Hungary; 3 2nd Department of Pathology, Semmelweis University, Budapest, Hungary; 4 1st Department of Surgery, Semmelweis University, Budapest, Hungary; 5 Biological Institute, Eötvös Loránd University, Budapest, Hungary

**Keywords:** Papillary thyroid cancer, New hotspot panel, Multivariate statistical method, Risk score, Next generation sequencing

## Abstract

Nowadays, the complementary diagnostics based on the suspicious thyroid lesion specific mutational state analysis is indispensable in the clinical practice. We aimed to test and validate our novel 568-mutational hotspot panel (23 cancer-related genes) on papillary thyroid cancers (PTCs) and their tumor-free pairs to find the most powerful mutation pattern related to PTC. The sequencing method was carried on with Ion Torrent PGM on 67 thyroid tissue samples. The most commonly detected mutation was the *BRAF* c.1799 T > A in all non-classical PTC cases. We utilized a multivariate statistical method (CVA) to determine a discrimination score based on mutational data array and to assess malignancy risk. Based on variants, the *BRAF* gene has by far the highest indicative power, followed by *TSHR* and *APC*. We highlighted novel aspects of the mutational profile and genetic markers of PTC. CVA has correctly assigned most of the samples based on the mutation frequencies and different variables of the selected genes, with high analytical probabilities. The final goal is to set up a new comprehensive rule-in and rule-out test to support the clinical decision making mainly in inconclusive fine-needle aspiration biopsy cases.

**Electronic supplementary material:**

The online version of this article (10.1007/s12253-019-00772-4) contains supplementary material, which is available to authorized users.

## Introduction

Thyroid cancer found in nodules of the gland is the most common endocrine malignancy (approximately 2.0–3.0% of all new cancers diagnosed each year in the USA) and its incidence is continuously growing worldwide during the recent decades [1]. The vast majority (60–80%) of thyroid cancers are well-differentiated papillary thyroid carcinomas (PTC) [2]. PTC tissues often dispose somatic mutations in the MAPK pathway elements, namely *BRAF*, *HRAS*, *KRAS* and *NRAS*, as well as in one of the most important tumor suppressor *TP53* gene. *BRAF* c.1799 T > A (V600E) is considered the most prevalent genetic alteration in PTC, and it has also been associated with more aggressive tumor behavior, such as extrathyroidal extension, lymph node involvement, resistance to radioactive iodine and tumor recurrence. Beside these common oncogenic mutations, rearrangements of *PAX8/PPARG* and *RET/PTC* genes are also notable in PTC variants [3]. Approximately 70% of differentiated thyroid tumors demonstrate one abnormalities of the four driver genes or either one of two chromosomal rearrangements [4]. In addition, newly identified genomic alterations have been implicated in the pathogenesis of differentiated thyroid cancers. These are also involved e.g. in *AKT* regulation (*PIK3CA*) and tumor suppression (*PTEN*). Simultaneous testing of the well-known driver genes and the novel ones combined with the histological findings facilitate more accurate clinical results. Furthermore, this approach might have potential usage in the complementary diagnostics of suspicious thyroid lesions.

The prevalence of palpable nodules in the thyroid gland is 4–7% [5], however, non-palpable and non-visible nodules are more frequent, altogether 20–50% of the population are affected depending on the method used to detect [6]. Majority of malignant lesions can be identified in cold nodules, i.e. nodules not taking up radioiodine. Fine needle aspiration cytology (FNAC) is the gold standard for the examination of different nodules. However, 10–40% of the biopsy cases will provide indeterminate diagnostic results: i, Bethesda III, atypia of undetermined significance/follicular lesion of undetermined significance (AUS/FLUS); ii, Bethesda IV, follicular neoplasm/suspicious for follicular neoplasm (FN/SFN); and iii, Bethesda V, suspicious for malignancy (SM) result [7, 8]. Recent studies [9, 10] showed that precancerous cytology completed with genetic testing may give a more precise diagnosis. Moreover, some somatic aberrations found in benign nodules could change the clinical decision making [8].

Tissue specific somatic mutation pattern analyses need sensitive, high-throughput, robust and cost-effective technology. Next generation sequencing (NGS) is capable of analyzing a huge number of genetic hotspots with as low as 5% allele frequency in one step. Recently, several multi-gene panels have been developed for thyroid tissue examination [11–13]. Our group established a novel generation of targeted sequencing panel for multiple mutational profiling in genes demonstrating significant role in thyroid carcinogenesis. This extended panel contains genetic variations that may lead to activation of the MAPK/MET/PI3K-AKT pathways or protooncogenes, as well as compromise the tumor suppressor function, based on a number of recent discoveries, including those reported by the National Cancer Genome Atlas Research Network in thyroid cancer cases [4]. In this work, we aimed to retrospectively test and validate our 568-mutational hotspot panel on PTC samples and their tumor-free pairs to find the most powerful mutation pattern related to papillary cancer. The final goal is to set up a new comprehensive rule-in and rule-out test to support the clinical decision making mainly in inconclusive FNAB cases, based on these preliminary results.

## Materials and Methods

### Biological Samples and Genomic DNA Isolation

In our present study, we analyzed genomic DNA from freshly frozen intraoperative samples of primary papillary thyroid carcinomas with matched tumor-free tissues. Samples were collected from patients undergo thyroidectomy at 1st Department of Surgery, Semmelweis University, between 2010 and 2016. All surgically removed thyroid tissue samples underwent detailed histological examination, and papillary carcinoma was identified in all cases. We excluded such samples when thyroid cancer was considered as metastatic secondary tumor. We examined 67 thyroid tissue samples, 39 with PTC and 28 tumor-free thyroid tissues. There were 27 sample pairs, i.e. both PTC and tumor-free thyroid tissues originated from the same patient, and 12 PTCs and 1 tumor-free tissue sample from different patients. Thus, samples were obtained from 40 patients. The study was approved by the National Committee for Research Ethics, and all patients gave written informed consent.

Intraoperative thyroid tissues were stored at −80 °C until processed. The samples were homogenized in phosphate-buffered saline (PBS) by Fisher Scientific Power Gen tissue grinder (Fisher Scientific GmbH, Germany). From each sample (tumor-free and cancerous), genomic DNA was separated by Roche High Pure PCR template Preparation Kit (Roche Indianapolis, IN, USA) according to the manufacturer’s protocol. The concentration of the isolated gDNA was determined using the Qubit dsDNA HS Assay Kit (Thermo Fisher, Waltham, MA, USA) and the quality was assessed using NanoDrop spectrophotometer (NanoDrop Technologies, Montchanin, DE, USA) at 260/ 280 nm. Our samples were characterized by LightCycler melting curve analysis for BRAF codon 600, KRAS codons 12 and 13, NRAS codon 61 and HRAS codon 61 [2].

### Ion Torrent Sequencing

A custom-made AmpliSeq hotspot panel was designed, using the AmpliSeq Designer software (Thermo Fisher, Waltham, MA, USA), targeting the relevant parts of coding sequences of 23 cancer-relate genes (*AKT1, APC, AXIN1, BRAF, C16orf3, CTNNB1, DICER1, EIF1AX, GNAS, HRAS, IDH1, KRAS, LPAR4, MET, NRAS, PIK3CA, PTEN, RET, SMAD4, TERT, TP53, TSHR, VHL*) which contains 568 known mutational hotspot areas with COSMIC IDs. The design process resulted in a total of 101 amplicons. To gain a higher coverage of the regions of interest, we designed the primers to flank some parts of the nearby introns, as well. Amplicon library was prepared using the Ion AmpliSeq Library Kit 2.0 (Thermo Fisher); briefly, multiplex primer pools were added to 10 ng of genomic DNA and amplified with the following PCR cycles: at 99 °C for 2 min, at 99 °C for 15 s, and at 60 °C for 4 min (21 cycles), and holding on at 10 °C. Primers were partially digested using a FuPa reagent, and then sequencing adapters were ligated to the amplicons. The library was purified in multiple times using the Agencourt AMPure XP Reagent (Beckmann Coulter, CA, USA). The concentration of the final library was determined by fluorescent measurement on Qubit 2.0 instrument (Thermo Fisher). Template preparation was performed with Ion PGM™ Hi-Q View OT2 kit (Thermo Fisher) on semiautomated Ion OneTouch 2 instrument using an emPCR method. After breaking the emulsion, the nontemplated beads were removed from the solution during the semiautomated enrichment process on Ion OneTouch ES (Thermo Fisher) instrument. After adding the sequencing primer and polymerase, the fully prepared Ion Sphere Particle (ISP) beads were loaded into an Ion 314v2 BC sequencing chip. Four samples loaded in one chip. The sequencing runs were performed using the Ion PGM™ Hi-Q™ View Sequencing kit (Thermo Fisher) with 500 flows.

### Data Analysis

Data from the Ion Torrent runs were analyzed using the platform-specific pipeline software Torrent Suite v5.0.4 for base calling, trim adapter and primer sequences, filtering out polyclonal and poor-quality reads, then de-multiplex the reads according to the barcode sequences. Briefly, TMAP (https://github.com/iontorrent/TMAP) algorithm was used to align the reads to the hg19 human reference genome, and then, the variant caller plug-in was selected to run to search for somatic variants in the targeted regions. Ion Reporter 5.0 was used for variant annotation. Variants with more than 1% population frequency were removed from the datasets. Alterations which are covered at least with 5 reads and presents in both strands were selected for further analysis. Integrative genomics viewer (IGV) was used for visualization of the mapped reads. We assessed the pathogenicity of the variants with SIFT and PolyPhen-2 algorithms.

### Canonical Variates Analysis

Data were evaluated at two levels, those of the variants and the genes. In the first case, we compiled a loci by patients matrix with 59 rows (identified variants on 18 genes) and 67 columns (samples) in which 0 indicated no mutation and 1 corresponded to mutation. This matrix was very sparse because most variants were detected in only one or two samples which have very low indicator value. We therefore reduced the data matrix to 26 by 67 by retaining those variants which occurred at least three times in the data. In the second case, the genes by patients data matrix of size 18 by 67 was generated by counting the number of variants detected for each gene in each patient.

The objective of our study was to select variants and genes that may be best used to predict the tumorous or benign state of the thyroid. Both data matrices were used as a training set in canonical variates analysis (CVA, alias discriminant analysis). Since the data obviously do not meet the statistical requirements for hypothesis testing, our goal was mainly exploratory. In other words, CVA is robust enough to give rough estimates of classification probabilities, while the use of canonical functions to classify unknowns was not warranted. Since the number of groups was 2, the analysis resulted in a single canonical variate for both sets of data. Correlations between the canonical variates and the original variables (26 variants or 18 genes) were calculated as a measure of their discriminatory – or tumor-indicative - power in separating the groups. For the purpose of estimating tumor risk, we formally used the sum of F-values (the ratio of between-group and within-group variances for each variable) derived from the training set: the larger this empirical value, the higher the risk. Due to the aforementioned statistical problems, no probability level could be associated to this statistic. We used the SYN-TAX 2000 package for computations [14].

## Results

### Descriptive Statistics

Nine males and 31 females participated in the study. The mean age of men was 55.1 years (range: 37–80 years) and in case of women was 49.5 years (range: 27–80 years). The papillary thyroid cancer samples were classified into seven histological subtypes. Twenty-four samples were recognized as classical PTC and 15 were different PTC variants as follicular (*n* = 4), encapsulated (*n* = 3), oncocytic (n = 4), multifocal type (*n* = 1) and microcarcinoma (n = 3). The largest tumor size occurred in follicular PTC variants (avg. diameter = 18.8 mm). Tumorous lesions affected both thyroid lobes beside multifocal type of PTC in case of classical and encapsulated variants. The basic characteristics of the specimens were shown in Table 1.

**Table 1:** The basic characteristics of the specimens

PTC samples (*n* = 39)	Male/Female (n)	Lobe involvement one/both (n)	Average tumor diameter (mm)	Lymph node metastasis (n)
Classical PTC (*n* = 24)	7/17	18/6	14.8	9/24
Follicular variant (*n* = 4)	1/3	4/0	18.8	1/4
Encapsulated variant (*n* = 3)	0/3	1/2	14.3	0/3
Oncocytic variant (*n* = 4)	0/4	4/0	16.3	3/4
Multifocal variant (*n* = 1)	0/1	0/1	N/A	0/1
Microcarcinoma (*n* = 3)	0/3	3/0	1.3	0/3

The 39 PTC and 28 tumor-free thyroid tissues were collected from 40 patients. More than a half of the thyroid samples categorized as classical PTC (*n* = 24) and the remaining 15 specimens belonged to other histological variants

### Quality Assessment of the Targeted Sequencing Data

The average read number was 143.211 reads per sample. The average base coverage depth was 642 (121–2.290) (Suppl. Table 1) and the average 1× target coverage was about 98.2% with a mean raw accuracy over 99%. The uniformity of coverage was between 54.4 and 93.2%.

### Mutational Profiling of PTC Samples Compared to Tumor-Free Tissue

Twenty-three relevant thyroid and thyroid cancer-related genes were tested on 568 known mutational hotspot areas in our 67 samples. Altogether 61 non-synonymous variants (36 SNVs: 35 missense and 1 nonsense; 25 INDELs: 24 insertions and 1 non-frameshift deletion) detected met the criteria concerning variant analysis (covered at least with 5 reads and presents in both strands). From all genetic alterations found, 31 were previously reported in COSMIC database. We were able to identify additional 30 novel variants surrounding the targeted hotspots. We excluded those variants for further analyses which occurred simultaneously in the tumor and its normal tissue pair.

In the tumorous tissues, we noticed 35 somatic mutations in 15 genes. The most polymorphic genes were *APC* and *BRAF* with 13–13 different variants, then *AXIN1* and *PIK3CA* represented with 5 and 4 single nucleotide variants (SNVs), respectively. The most commonly detected mutation was the well-known *BRAF* c.1799 T > A (V600E) in 13 PTC samples. Other frequently identified variants were the formerly uncharacterized *TSHR* c.1373 T > C alteration in four cancer samples and the *APC* c.636_637insAA frameshift mutation in four tumor tissues as well (Suppl. Table 2). We assessed the pathogenicity of the variants with SIFT and PolyPhen-2 algorithms and the results can be found at supplementary Table 2.

On average, 2.56 mutations (range: 0–13) were recognized per thyroid cancer sample. Thirteen nucleotide changes were the highest number that was harbored in one PTC specimen, and nine tumors showed no somatic mutation in the analyzed genes. PTC samples containing *BRAF* V600E had lower average mutation density (AMD =2.9) than tumorous tissues without *BRAF* mutation where AMD was 3.7.

Aberrations of *GNAS* as a benign thyroid nodule indicator were not detected in any of the PTC samples. The mutational landscape of our study samples did not show any of the well-characterized *TERT* promoter alterations.

Compared to the tumor samples, mutation density was nearly a third in tumor-free benign thyroid tissues. In total, 11 mutations were determined in 7 genes. *LPAR4* was the most variable gene with six different variants. The most prevalent mutation was *LPAR4* c.137A > G which appeared in four samples. In case of 15 genes (*AKT1, BRAF, DICER1, GNAS, HRAS, KRAS, MET, NRAS, PIK3CA, RET, SMAD4, TERT, TP53, TSHR* and *VHL*), somatic alterations were not detected. We considered a variation to be likely pathogenic if it causes a frameshift in tumor suppression gene or it is classified as deleterious by both SIFT and PolyPhen-2 algorithms. Identified likely pathogenic mutations from normal tissue may be present in cells which are already carriers of novel deleterious alterations but remains undetected during surgery. Three of the 11 variations were not damaging based on SIFT and PolyPhen-2 softwares, and 5 of the 11 alterations in benign samples were frameshift mutations. The remaining 3 variants proved to be damaging by both algorithms.

The LightCycler melting curve analysis was carried out for *BRAF* codon 600, *KRAS* codons 12 and 13, *NRAS* codon 61 and *HRAS* codon 61, and the concordance was 100% with our NGS based study.

### Multivariate Classification of Genomic Data to Estimate Malignancy Risk Score

#### Discriminant Scores for the Variants by Samples Data

**Fig. 1:**
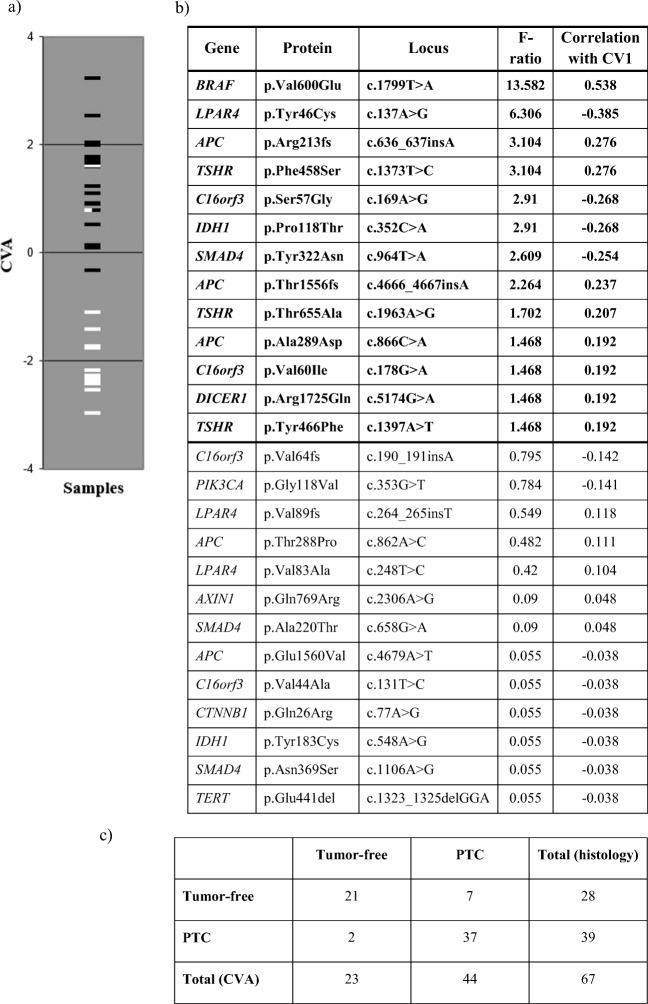
The relationship between the actual state of the tumor-free and PTC samples, analysis of the selected 26 variants which occurred more than twice or more times. **a** Graphical display of samples separated by CVA of the selected 26 variants which were determined more than 2 or more times in our cohort. The benign tumor-free samples are marked with white lines and PTC samples are represented by black lines. **b** Table is summarizing the calculated F-ratios and the correlations with the single canonical variate (CV1). F-ratio derived from the training set: the larger this empirical value, the higher the malignancy risk. **c** Our histologically confirmed PTC or tumor-free samples were grouped according to the 26-variant set. Twenty-one benign samples out of the total 28 were actually assigned into the tumor-free group. Thirty-seven patients with PTC out of the total 39 were considered as malignant

As mentioned earlier, the first analysis was based on the 26 variants which occurred at least three times in the samples. The F-ratios and correlations with the single canonical variate are given in descending order of F-values in Fig. 1. Since the two groups of patients separate fairly well along this axis, we can safely say that positive correlations refer to markers indicating the presence of tumor, while negative correlations indicate the condition of tumor-free thyroid tissue.

The *BRAF* c.1799 T > A (V600E) point mutation had the greatest indicative power of all variants; actually this mutation was present in 13 tumors and never occurred in tumor-free tissues. There are further twelve alterations with F > 1 that may also be included in the group of target variables (Fig. 1b.). Those with F < 1 are not useful as indicators, their occurrence in patients appears to be random. Thus, genetic analysis could be useful even if it is restricted to the following 13 variants (calculating the sum of F values for variants with the absolute value of correlations higher than 0.136): *BRAF* c.1799 T > A, *LPAR4* c.137A > G, *APC* c.636_637insA, *TSHR* c.1373 T > C, *C16orf3* c.169A > G, *IDH1* c.352C > A, *SMAD4* c.964 T > A, *APC* c.4666_4667insA, *TSHR* c.1963A > G, *APC* c.866C > A, *C16orf3* c.178G > A, *DICER1* c.5174G > A and *TSHR* c.1397A > T.

Since patients with no mutation in the above listed 13 variants took a value of 0.136 or smaller on the single axis, we can consider all patients with a score ≤ 0.136 as being classified by the method as tumor-free, all others cancerous. The cross-classification table showing the relationship between the actual status of the patients (tumor-free vs cancerous) and the assignment of the patients into either group based on the 26 variables is shown in Fig. 1c.

Actual status and group assignment are not independent: most of tumor-free patients were actually assigned into the healthy group, and most of patients diagnosed with tumor were assigned to the cancerous group. We thus estimated the following probabilities: sensitivity: 37/39 = 0.94; specificity: 21/28 = 0.75; positive predictive value: 37/44 = 0.84; negative predictive value: 21/23 = 0.91.

#### Discriminant Scores Based on Mutation Frequencies of Genes

**Fig. 2:**
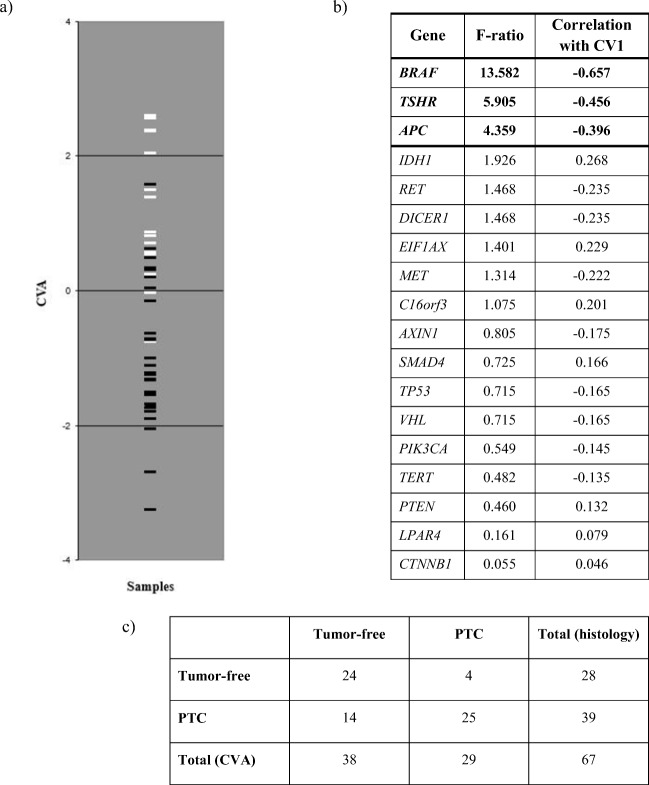
The relationship between the actual state of the tumor-free and PTC samples, analysis of the selected 18 genes based on mutation frequencies. **a** Graphical display of samples separated by CVA of the selected 18 genes where alterations were detected. The benign tumor-free samples are marked with white lines and PTC samples are represented by black lines. **b** Table is summarizing the calculated F-ratios and the correlations with the single canonical variate (CV1). F-ratio derived from the training set: the larger this empirical value, the higher the malignancy risk. **c** Our histologically confirmed PTC or tumor-free samples were grouped according to the 18-gene set. Twenty-four benign samples out of the total 28 were actually assigned into the tumor-free group. Twenty-five patients with PTC out of the total 39 were considered as malignant

CVA was also performed on the 18 genes from the initial set for which alterations (at least one variant or mutation) were detected. The F-ratios and correlations with the single canonical variate for the 18 genes are given in Fig. 2 in descending order of F-values. Apparently, correlations larger than 0.322 indicate presence of tumors whereas others reflect the tumor-free state.

In agreement with the analysis based on variants, the *BRAF* gene has by far the highest indicative power, followed by *TSHR* and *APC.* The first gene in which mutations tend to appear mostly in histologically benign thyroid is *IDH1*. Then, further 6 genes (*EIF1AX*, *C16orf3*, *SMAD4*, *PTEN*, *LPAR4* and *CTNNB1*) follow which have an F value >1. Those with lower F have definitely no indicative power in detecting tumor. The empirical summary statistics for unknowns may be calculated for genes as above.

Since the patients completely free of mutation had a score of 0.322, we can consider all patients with a score ≤ 0.322 as being classified by the method as tumor-free, all others cancerous (Fig. 2b.). The cross-classification table showing the relationship between the actual status of the patients (tumor-free vs cancerous) and the assignment of the patients into either group based on the 18 genes is shown in Fig. 2c.

As in case of variants, CVA has correctly assigned most of the patients based on the mutation frequencies in the 18 genes: most of tumor-free patients were actually assigned into the healthy group, and most of patients diagnosed with tumor were assigned to the cancerous group. We thus estimated the following probabilities in this case: sensitivity: 25/39 = 0.64, specificity:24/28 = 0.86; positive predictive value: 25/29 = 0.86; negative predictive value: 24/38 = 0.63.

In this work, we have assembled and validated a 23-gene-based, targeted, massively paralleled sequencing assay for the auxiliary diagnosis of papillary thyroid cancer cases. Our hotspot panel using rule-in / rule-out approach includes genetic mutations that are considered to be markers of aggressive tumor behavior. We have firstly utilized a multivariate statistical method (CVA) to determine a discrimination score based on mutational data array and to assess malignancy risk. With this approach, we have successfully identified the large majority of malignant as well as benign cases.

According to the NGS results the most commonly detected mutation was the well-known driver *BRAF* c.1799 T > A (V600E) in 13 PTC samples. Based on recent literature, the *BRAF* V600E variant occurs in approximately 45% of PTCs [15]. *BRAF* is a member of the RAF kinase family that promotes signaling through the RAS/RAF/MAPK signal-transduction cascade. Expression of BRAF mutant protein causes the constitutive activation of the pathway which sustains tumor growth and is associated with poor clinical outcome. The *BRAF* V600E activating mutation accounted for all the examined histological subtypes of PTC tissues. Our results highlight the possibility that defining *BRAF* genotypes surrounding V600E hotspot may have significant clinical consequences.

It is generally believed that genetic alterations in RAS family and *BRAF* are mutually exclusive in PTC [16]. There were no *RAS* mutation in the 13 *BRAF* V600E positive thyroid cancers. *KRAS* mutations known to cause only benign thyroid neoplasia were not detected in our PTC samples.

The *APC* was the most variable oncogene with 13 mutations found in PTC samples. It is an important key regulatory element of WNT pathway. Loss of functional APC protein results in activation of canonical WNT/β-catenin signaling and initiates carcinogenesis [17]. The *APC* gene was initially identified as being causative for the familial adenomatous polyposis (FAP) syndrome [18]. Sporadic *APC* mutations are reported to be associated with a rare cribriform-morular variant of papillary thyroid carcinoma in FAP, but they are also encountered in non-FAP patients [19]. Furthermore, *APC* mutations have been observed in anaplastic thyroid carcinomas as well [20]. Recent studies on PTC exome DNA sequence data denoted *APC* as a potential driver [4]. Similarly, in our NGS results, *APC* had strong discriminative power and its alterations correlated with malignant transformation.

AXIN1 has been classically thought to be a tumor suppressor protein. Activation of the WNT signaling pathway by alterations in the signaling components, such as AXIN1 leads to accumulation of β-catenin which translocates to the nucleus and modulates the expression of target genes [17]. Altogether, 6 genetic alterations of *AXIN1* were detected in our PTC and tumor-free samples, however, the mutational patterns were markedly different. In PTCs, 2 INDEL, 1 nonsense and 2 missense variants were identified. Variant derived exclusively from normal thyroid tissue was classified as missense. One variant, namely, c.2306A > G, out of the all *AXIN1* variants was common in both cancerous and benign thyroid. Based on our discrimination scoring method, *AXIN1* correlated with tumorous state, however, only with low F ratio. At present, there is only limited information on *AXIN1* abnormalities that were mostly reported in poorly differentiated thyroid cancers (e.g. anaplastic form) [21]. Its potential role in the development and behavior of well-differentiated thyroid carcinomas have not yet been established.

Increased mitogenic signal via PI3K/AKT cascade has a fundamental role in thyroid tumorigenesis [22]. Mutations of *PIK3CA*, which encodes the p110α catalytic subunit of PI3K, are common in thyroid cancer. In agreement with the current literature, our multivariate analysis based on the presence of *PIK3CA* gene alterations exhibited it as a reliable indicator of cancerous state. On the other hand, genetic alterations involving *PIK3CA, AKT1* and *TP53* are rarely present in benign thyroid nodules [8]. One study of Pan et al. [23] demonstrated that variants of the PI3K/AKT pathway genes (i.e. *PIK3CA, PIK3R2, AKT2, PTEN* and *TP53*) seemed to be mutually exclusive for thyroid cancers and affected 12.1% of Chinese PTC patients. This was also confirmed by our results, our PTC samples didn’t show coexistence of different PI3K/AKT pathway gene mutations.

The PI3K/AKT pathway is known to have a close crosstalk with the RAS/RAF/MAPK signaling cascade. Numerous PTCs with *PIK3CA* alterations have been demonstrated to be mutated in *RAS* or at *BRAF* genes as well [22]. Our results support this observation since we found 2 PTCs with simultaneous *BRAF* and *PIK3CA* mutations.

Several previous studies have examined the role of the promoter mutations in the *TERT* gene (C228T and C250T) in thyroid cancer [24, 25]. The data so far indicate that *TERT* promoter mutations are infrequent in PTC but have been identified as an indicator of the bad prognosis [26]. The mutational landscape of our study samples did not show any of the well-characterized *TERT* promoter alterations. The diagnostic and prognostic utility of *TERT* promoter mutations in papillary neoplasm are controversial and requires additional investigations.

In summary, our NGS analysis corroborates the recent findings that papillary thyroid cancer is genetically more diverse than previously recognized [4]. Several from our conclusions are in accordance with the current literature, however, we highlighted novel aspects of the mutational profile and genetic markers of PTC. We have firstly utilized a multivariate statistical method to determine a discrimination score based on mutational data array and to assess malignancy risk. Retrospectively, CVA has correctly assigned most of the samples based on the mutation frequencies and different variables of the selected genes, with high analytical probabilities. Our NGS method for the auxiliary diagnostics of thyroid nodule samples for PTC might give rise of a new comprehensive rule-in and rule-out test to support the clinical decision making, mainly in inconclusive FNAB cases. Using next-generation sequencing is a cost-effective and high throughput capacity technique which could be successfully integrated into clinical routine. Further studies are required to adopt our targeted NGS-based, multivariate test for inhomogeneous fine-needle aspiration biopsy samples.

## Electronic supplementary material

ESM 1 (DOCX 19 kb)

## Abbreviations

AUS/FLUS: Atypia of undetermined significance/follicular lesion of undetermined significance
COSMIC: Catalogue Of Somatic Mutations In Cancer
CVA: Canonical variates analysis
DNA: Deoxyribonucleic acid
FNAB: Fine needle aspiration biopsy
FNAC: Fine needle aspiration cytology
FN/SFN: Follicular neoplasm/suspicious for follicular neoplasm
IGV: Integrative genomics viewer
INDEL: Insertion-deletion polymorphisms
PBS: Phosphate-buffered saline
PTC: Papillary thyroid cancer
SM: Suspicious for malignancy
SNV: Single nucleotide variant
NGS: next generation sequencing
