# Supplementary material for: Targeted Mutational Profiling and a Powerful Risk Score as Additional Tools for the Diagnosis of Papillary Thyroid Cancer
Source: Pathol Oncol Res. 2019 Nov 22;26(1):101–8. doi: 10.1007/s12253-019-00772-4 (PMC7109166; doi:10.1007/s12253-019-00772-4)
Supplement: Supplementary file 1 — (DOCX 19 kb) [file 12253_2019_772_MOESM1_ESM.docx]

**Suppl table 1. Amplicon based coverage per sample (attached)**

A few of our samples had only poor quality of DNA and this type of samples produced lower uniformity values. We generated more sequencing reads for poor quality samples to achieve enough coverage for variant calling. However, there are certain amplicons in certain samples which could not gained enough coverage for high sensitivity variant calling. We had four samples these had low library concentration (between 50 and 100 pM). We evidently accepted library concentrations over 100 pM for sequencing, while we excluded those samples <50 pM concentration. If concentrations were between 50 and 100 pM but the DNA quality was good, we went ahead and sequenced the sample. The library concentrations of these 4 samples fell in such category.

**Suppl table 2. Mutations found in 20 genes specific only for PTC samples.**

| **Gene** | **COSMIC ID** | **Locus** | **Protein** | **Allelic frequency of detected mutations (%)** | **SHIFT** | **PolyPhen-2** | **Number of tumor samples**  **(classical PTC / PTC subtypes)** |
| --- | --- | --- | --- | --- | --- | --- | --- |
| APC |  | c.636_637insAA | p.Arg213fs | 5.03  5.67  12.14  13.57 | - | - | 4/0 |
| APC | COSM18561 | c.4666_4667insA | p.Thr1556fs | 6.47  7.69  8.65 | - | - | 3/0 |
| APC | COSM19119 | c.4364_4365insA | p.Asn1455fs | 5.03 | - | - | 1/0 |
| APC |  | c.4478_4479insA | p.Glu1494fs | 10.1 | - | - | 1/0 |
| APC |  | c.3022_3023insA | p.Ile1008fs | 6.59 | - | - | 1/0 |
| APC |  | c.4778_4779insA | p.Pro1594fs | 5.90 | - | - | 1/0 |
| AXIN1 |  | c.1900C>T | p.Gln634Ter | 5.34 | - | - | 1/0 |
| AXIN1 |  | c.1918_1919insGA | p.Glu640fs | 11.90 | - | - | 0/1 |
| AXIN1 | COSM5752849 | c.1326_1327insC | p.Ala443fs | 5.26 | - | - | 1/0 |
| AXIN1 |  | c.1655C>T | p.Ala552Val | 5.49 | 0.1 | 0.978 | 1/0 |
| AXIN1 |  | c.2155A>G | p.Lys719Glu | 23.07 | 0.01 | 0.605 | 0/1 |
| BRAF | COSM476 | c.1799T>A | p.Val600Glu | 10.42  11.94  23.30  24.24  24.53  26.09  26.99  33.75  33.78  34.78  40.91  41.66  42.19 | 0 | 0.971 | 9/4 |
| C16orf3 |  | c.178G>A | p.Val60Ile | 6.86  11.86 | - | - | 2/0 |
| DICER1 | COSM4169490 | c.5174G>A | p.Arg1725Gln | 5.19  7.04 | 0.05 | 1 | 1/1 |
| LPAR4 |  | c.257T>C | p.Leu86Ser | 5.58 | - | - | 1/0 |
| LPAR4 |  | c.208_209insA | p.Met70fs | 8.30 | - | - | 1/0 |
| MET |  | c.1601C>A | p.Pro534His | 6.45 | 0.08 | 0.483 | 1/0 |
| MET |  | c.3075_3076insT | p.Pro1026fs | 5.16 | - | - | 0/1 |
| MET | COSM707 | c.3029C>T | p.Thr1010Ile | 50.72 | 0.06 | 1 | 0/1 |
| PIK3CA | COSM763 | c.1633G>A | p.Glu545Lys | 13.46 | 0 | 0.991 | 1/0 |
| PIK3CA |  | c.2063_2064insT | p.Leu688fs | 6.33 | - | - | 0/1 |
| PIK3CA |  | c.975_976insA | p.Ser326fs | 5.88 | - | - | 1/0 |
| PIK3CA | COSM17444 | c.3061T>C | p.Tyr1021His | 7.74 | 0 | 0.993 | 1/0 |
| PTEN |  | c.416_417insTT | p.Leu139fs | 5.88 | - | - | 1/0 |
| RET | COSM965 | c.2753T>C | p.Met918Thr | 41.01 | 0 | 0.999 | 1/0 |
| RET |  | c.2185_2186insA | p.Thr729fs | 5.05 | - | - | 1/0 |
| SMAD4 |  | c.907C>T | p.Pro303Ser | 9.35 | 0.37 | 0.017 | 1/0 |
| TERT |  | c.1262_1263insC | p.Ala422fs | 5.03 | - | - | 1/0 |
| TERT |  | c.1374_1375insC | p.Trp459fs | 7.46 | - | - | 1/0 |
| TP53 | COSM43533 | c.391A>T | p.Asn131Tyr | 23.33 | 0 | 1 | 1/0 |
| TSHR |  | c.1373T>C | p.Phe458Ser | 5.91  5.94  7.34  11.16 | 0 | 0.995 | 2/2 |
| TSHR |  | c.1397A>T | p.Tyr466Phe | 5.59  10.94 | 0 | 0.999 | 2/0 |
| TSHR |  | c.1864_1865insAA | p.Ile622fs | 8.42 | - | - | 1/0 |
| VHL |  | c.158A>G | p.Glu53Gly | 5.5 | 0.02 | 0 | 1/0 |
